# Bioactive Compounds Derived from the Yeast Metabolism of Aromatic Amino Acids during Alcoholic Fermentation

**DOI:** 10.1155/2014/898045

**Published:** 2014-05-05

**Authors:** Albert Mas, Jose Manuel Guillamon, Maria Jesus Torija, Gemma Beltran, Ana B. Cerezo, Ana M. Troncoso, M. Carmen Garcia-Parrilla

**Affiliations:** ^1^Facultad de Enología, Universitat Rovira i Virgili, Marcel*·*lí Domingo s/n, 43003 Tarragona, Spain; ^2^Departamento de Biotecnologia de Alimentos, Instituto de Agroquímica y Tecnología de los Alimentos (CSIC), Agustín Escardino, 7, 46980 Valencia, Spain; ^3^Facultad de Farmacia, Universidad de Sevilla, Profesor García González, 2, 41012 Sevilla, Spain

## Abstract

Metabolites resulting from nitrogen metabolism in yeast are currently found in some fermented beverages such as wine and beer. Their study has recently attracted the attention of researchers. Some metabolites derived from aromatic amino acids are bioactive compounds that can behave as hormones or even mimic their role in humans and may also act as regulators in yeast. Although the metabolic pathways for their formation are well known, the physiological significance is still far from being understood. The understanding of this relevance will be a key element in managing the production of these compounds under controlled conditions, to offer fermented food with specific enrichment in these compounds or even to use the yeast as nutritional complements.

## 1. Nitrogen Metabolism during Alcoholic Fermentation

The transformation of grapes into wine is a biotechnological process where microorganisms, primarily yeast, convert a sugary liquid in a water-alcohol solution of flavour and pleasant aroma. To perform this process, they use the nutrients present in the medium for growth, producing a range of metabolites that yield the complexity of fermented beverage.

The grape must is a very complex food product, with a variety of compounds ranging from mainstream (sugars) to very small but important quantities, from both nutritional (vitamins, minerals, and polyphenols) and organoleptic (flavour and precursors) points of view. However, far from being an optimal culture medium, it is indeed a highly selective medium. This selectivity is due to the high sugar content, present in equimolar concentrations of glucose and fructose between 170 and 280 g/L, low pH (ranging from 2.8 to 3.5), nutrient limitation (especially nitrogen), and some technological practices such the addition of SO_2_ (up to 150 mg/L in certain cases) and fermentations with a broad range of temperatures (from 10°C up to 35°C). However, the sugar concentration can reach much higher concentrations in certain cases, such as dehydration and overripening. Overripening could be natural (raisins, attacks from* Botrytis* and other fungi) or induced during wine making (cooking must, water elimination by reverse osmosis, or using frozen grapes, etc.). Additionally, there is a strong imbalance with the nitrogen fraction, which is in a concentration three orders of magnitude lower (concentrations between 70 and 600 mg/L). This nitrogen component plays a predominant role in the fermentation process. Grape must contains a variety of nitrogen compounds, among which the most important are amino acids, ammonium ion, and small peptides. These nitrogen compounds, excluding proline, constitute what is called yeast assimilable nitrogen. Nitrogen affects yeast cells in two aspects: biomass production during fermentation and the fermentation rate [1]. Therefore, the nitrogen content exerts an action on fermentation by regulating both its rate and its end. In fact, the lack of nitrogen has been pointed as one of the main reasons of stuck or sluggish fermentations [2, 3]. Stuck and sluggish fermentations are detrimental for wine quality as they leave residual sugars that would increase microbial instability and change the organoleptical properties of the final wine. The nitrogen content also affects other pathways in yeast, in particular, through the redox status of the cells, which affects the production of ethanol and other metabolites such as glycerol, acetic acid, and succinic acid [4–6]. Finally, other metabolites very relevant to wine quality are the volatile compounds and* Saccharomyces cerevisiae* produces different concentrations of those depending on fermentation conditions. Among these conditions, the quality and quantity of the nitrogen sources are critical in the formation of some aromatic molecules [7]. A range of volatile compounds such as acetate and ethyl esters, higher alcohol, volatile fatty acids, and carbonyls, which are the main molecules contributing to secondary or fermenting wine flavour, are mainly synthesised as metabolites derived from the metabolism of nitrogen [7–10]. Thus, nitrogen availability modulates the organoleptic quality and the taste of wine [11].

The presence of nitrogen in any of these chemical forms is highly variable, depending on various factors, including grape variety, degree of ripeness, soil, climate characteristics, and various technological aspects (type of vinification, pressing, etc. [12]). The current context of global warming, which results in overripe grapes, has two very direct effects on the composition of the must: higher sugar concentration and lower nitrogen levels. This combination produces higher fermentation hurdles. Thus, the knowledge of the nitrogen needs of different wine yeast strains used in the wine industry becomes more necessary. The addition of nitrogen to must is a very common practice among winemakers to avoid fermentation problems. Thus, the nitrogen addition should be adjusted to the real needs of each wine strain to prevent excessive concentrations, which would have negative consequences. The most relevant ones are the microbial instability of wines due to the nitrogen availability for the proliferation of other microorganisms or the synthesis of unhealthy substances, such as ethyl carbamate synthesis formed by yeast or biogenic amines, due to lactic acid bacteria during the malolactic fermentation using this residual nitrogen. Therefore, there is a need to optimise the use of the nitrogen by wine yeast, leaving very limited amounts of amino acids. Furthermore, excess nitrogen has also an impact on the organoleptic characteristics, such as the production of ethyl acetate or fruity aromas, depending on the type of nitrogen added (inorganic or organic, [13])

Although nitrogen concentration is a relevant factor, it is also important to underline that not all the nitrogen sources support equally yeast growth. In complex mixtures of amino acids and ammonium, such as grape must, wine yeasts have preference for some nitrogen sources, and the pattern of the preferential uptake of the nitrogen sources is determined by different molecular mechanisms. In* S. cerevisiae*, the mechanism is known globally as nitrogen catabolite repression (NCR). The NCR allows cells to detect the presence of the best sources of nitrogen by limiting the use of those that do not allow for the best growth. The detection of the rich nitrogen sources triggers a signalling chain that culminates with the activation of genes involved in the transport and metabolism of these rich sources and the suppression of those genes involved in the transport and use of poorer sources. Once the richest sources of nitrogen (ammonium, glutamine, and asparagine) are consumed, yeast metabolism activates the utilisation of the poorer sources of nitrogen (arginine, glutamate, alanine, etc.). Gutiérrez et al. [7] quantified the effect of different nitrogen sources on the three main parameters related to yeast growth (lag phase, generation time, and population size) in four commercial wine yeasts widely used in Spanish wineries, obtaining significant differences in these parameters concerning both the strain and the nitrogen sources. However, this study concluded that the categorisation between “good” sources and “bad" sources was dependent on the carbon backbone resulting from the metabolism of these amino acids. The transamination or deamination of “good” sources, which support rapid cell growth, produces easily assimilable carbon compounds by cell metabolism. This is the case of high growth rate sources such as glutamine, asparagine, glutamate, or alanine, which produce carbonyl derivatives such as *α*-ketoglutarate or pyruvate, which are readily integrated into the yeast fermentative metabolism. Instead, the amino acids with complex carbon backbones, which need to be detoxified or go through a complex metabolism, support slower growth.

## 2. Derivatives of Aromatic Amino Acid Metabolism and Its Physiologic Consequences: The Ehrlich Pathway

**Figure 1:**
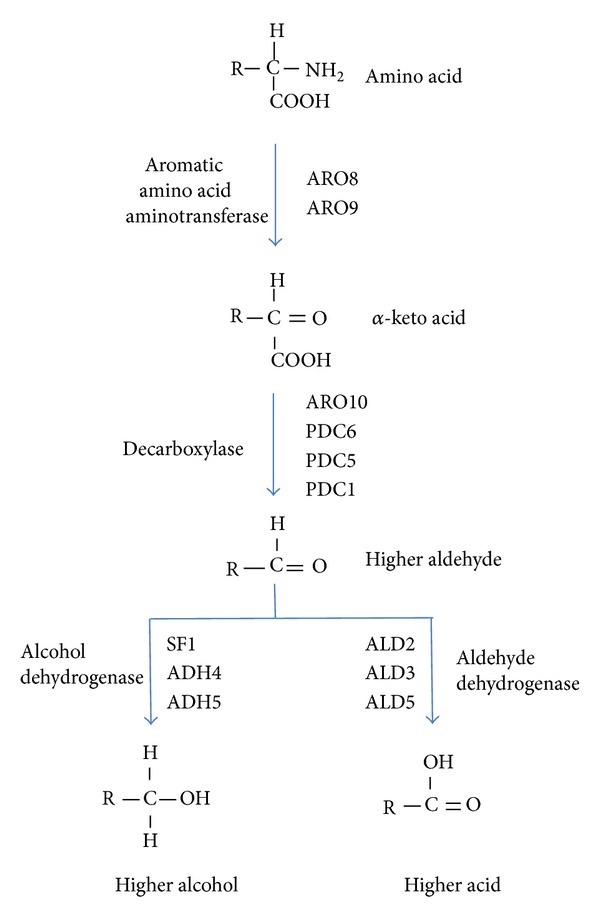
Ehrlich pathway of aromatic amino acids with indication of the enzymes and their coding genes.

Aromatic amino acids are catabolised by the Ehrlich pathway, which starts with the transamination of the amino group and the formation of *α*-keto acid (Figure 1), such as indole pyruvate, phenyl pyruvate, and 4-hydroxyphenyl pyruvate from tryptophan, phenylalanine, and tyrosine, respectively (Table 1). Subsequently, these keto acids are decarboxylated to the corresponding aldehydes (indole acetaldehyde, phenyl acetaldehyde, and 4-hydroxyphenyl acetaldehyde). Finally, depending on the redox state of the cell, they can be further metabolised to the corresponding aromatic alcohol, indole 3-ethanol (tryptophol), phenyl ethanol, and tyrosol, or are oxidised to the corresponding acids, indole acetic acid, phenyl acetic acid, and 4-hydroxyphenyl acetic acid.

As already mentioned, these higher alcohols affect wine aroma, especially 2-phenyl ethanol, which has a nice scent of roses, highly desired in some wines. Because of its industrial importance (it is widely used in cosmetics and as food additive), the production of this compound is well known among the full range of aromatic alcohols produced by yeast (for a review, see Hua and Xu [14]). Less attention has been given to the other types of aromatic alcohols, such as tryptophol or tyrosol, although their concentrations in some cases are also relatively high in wine (reaching up to 50 mg/L [10]). Regardless of the contribution of these higher alcohol levels to wine aroma, they have been recently described as the molecular modulators of some physiological and morphological processes considered involved in cell signalling. Hence, these higher alcohol levels have been linked with the stimulation of pseudohyphal growth in* S. cerevisiae*, resulting in a decrease in the growth rate [15]. This aspect has been also called as quorum sensing in yeast, which is related to population size and a morphological change from yeast to pseudohypha. The concept of quorum sensing appears in bacterial studies, described as the underlying mechanism that regulates the bacterial population in a variety of situations. In recent years, there have been several studies of quorum sensing in fungi and yeast species [16]. Interestingly, all these cases involve transient morphological changes of filamentous mycelium to yeast state or vice versa. One of the best-studied cases is the dimorphic human pathogenic yeast,* Candida albicans*. This yeast, depending on the conditions of the culture medium, moves from the nonpathogenic yeast form to the formation of hyphae or pathogenic form. Two alcohols that act antagonistically mediate this change: farnesol (intermediate in the synthesis of sterols) and tyrosol. Farnesol is excreted during cell growth, and when the cell population is high, the synthesis of farnesol increases and inhibits the formation of hyphae [17]. On the contrary, when the culture is diluted to a low cell density, the production of tyrosol promotes the formation of hyphae, and thus the yeast becomes a pathogen [18].

These morphological changes have also been observed in* S. cerevisiae* and also related to cell density signalling or quorum sensing. Chen and Fink [19] found that this yeast in stationary phase with high cell density and nutritional deficiencies, particularly in nitrogen, underwent pseudohyphal growth. Transcriptionally, this induction was associated with a fivefold increase in the activity of the* FLO11* gene, an essential gene for pseudohyphal growth [20]. However, this induction was biochemically linked to two types of aromatic alcohols: phenyl ethanol and tryptophol. The addition of these kinds of alcohol to cultures resulted in very invasive pseudohyphal growth, along with an increased induction of* FLO11*. The final proof of the involvement of these types of aromatic alcohols in this morphological change was that mutants in* ARO9* and* ARO10* genes, required for the synthesis of phenyl ethanol and tryptophol, dramatically decreased the pseudohyphal growth [19]. In turn, the expression of these genes is induced by tryptophol in a sort of self-stimulatory cycle. Therefore, high population densities produce more types of aromatic alcohols per cell compared with low population densities. These studies point to a direct relationship between the synthesis of these types of aromatic alcohols and the signalling cell density, nutrient deficiency, and entry into stationary phase.

However, the effect of these types of aromatic alcohols on humans is not only related to the flavour effect due to its presence in fermented foods. Tyrosol has been described as an antioxidant in human cell lines [21] and also as a cardioprotective agent [22]. The latter has been related to its presence in wine and attributed to some of the positive actions of moderate wine consumption [23]. On the other hand, tryptophol has been demonstrated to induce sleep in mice [24], although this action could be due to functional analogue or precursor of serotonin or melatonin.

## 3. Synthesis of Other Bioactive Compounds from Aromatic Amino Acids

**Figure 2:**
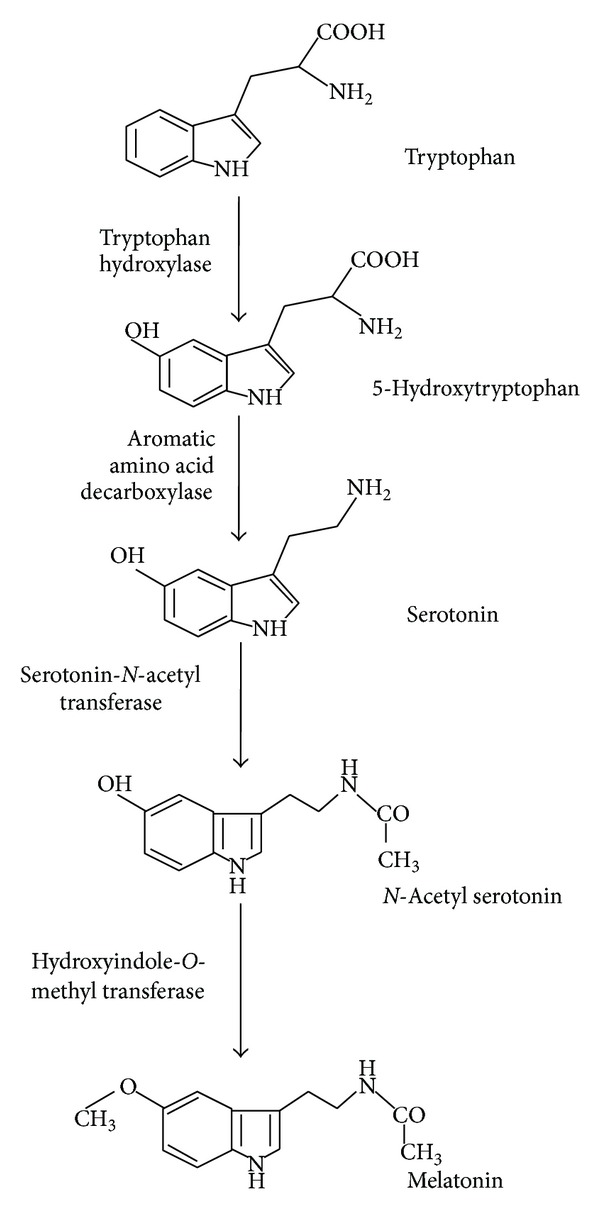
Synthesis of melatonin from tryptophan in yeast.

Moreover, there are other metabolites derived from these aromatic amino acids that are putative bioactive molecules with interesting properties. They have been only very recently described, and their metabolic pathways, regulation, coding genes, and so on are still under research. One of them is melatonin, which has been recently detected in wine, and its presence has been related to the activity of the yeast involved in the fermentation process [25, 26]. Originally, melatonin was seen as a unique product of the pineal gland of vertebrates and was called a neurohormone. However, in the last two decades, it has been identified in a wide range of invertebrates, plants, bacteria, and fungi. Therefore, today it is considered that melatonin is a ubiquitous molecule present in most living organisms [27]. Although little information is available on melatonin biosynthesis in organisms other than vertebrates, in yeast the pathway seems to be similar to the synthetic route and enzymes described in vertebrates [28]. This synthesis route is very simple, with four enzymes involved in the conversion of tryptophan to serotonin and* N*-acetylserotonin intermediates and finally to melatonin (Figure 2).

In humans, melatonin is a hormone that modulates physiological processes, such as circadian rhythms and reproductive functions, and also acts as an antioxidant [29–31]. In animals, melatonin typically occurs in the pineal gland (although subsequently described in other tissues and synthesised in important levels also in the intestine [32]), and its effects are very large, producing a pleiotropic response. Apart from the very well-described role as a regulator of circadian rhythm, melatonin has also been associated with antioxidant effects. These effects are not associated with a typical redox cycle but with a cascade of metabolites that turn into antioxidant activities [33, 34]. These antioxidant effects have also been correlated with an increased longevity [35] and the development of protective mechanisms against mutations [36], which would allow for a radioprotective effect [37]. Melatonin seems also to affect the immune system [38], although the mechanisms of action are poorly established. However, most of its effects suggest a clear neurohormonal activity, which has allowed us to relate their presence to learning and memory processes [39], ageing, and treatment for Alzheimer's disease [40, 41], amyotrophic lateral sclerosis [42], or migraine [43].

Regarding the presence or production of melatonin in yeast, the pioneer study of Sprenger et al. [28] related the presence of* S. cerevisiae* and the production of melatonin. Later, some reports detected melatonin in wines [44–46] and beer [47]. Recent studies also describe melatonin in grapes and other tissues of the vine, which could indicate that the origin was the substrate [23, 48, 49]. However, all the references that analyse the presence in wines and grape must indicate the production of melatonin during fermentation, being absent in the initial grape must [25, 47]. The description of melatonin in wine has linked its formation with yeast metabolism [25, 26], although the number of references in this case is still rare, indicating the need to pursue further this subject. In addition, all previous studies have focused exclusively on melatonin production by* Saccharomyces *yeasts, without considering the presence and metabolic activities of non-*Saccharomyces* wine yeast, significantly present in grapes and at the beginning of alcoholic fermentation. Therefore, the possible relation of the non-*Saccharomyces* wine yeast with the production of melatonin during alcoholic fermentation needs further evaluation.

Although the functions of melatonin are clear in mammals and animals [50], mainly related to regulatory mechanisms involved in circadian rhythms [51], the role of melatonin in yeast and other microorganisms seems to be even very far from being understood. Indeed, although the presence of circadian rhythms in yeast has been determined [52], this seems to be far from independent daily rhythms and regulated in response to the light produced in the multicellular organisms described. Instead, the response in yeast is induced by temperature changes only after several generations in chemostats and appears to be related to the primary nitrogen metabolism, particularly, to the expression of transporter genes of some nitrogen compounds (MEP2, which is the transporter on ammonium, and GAP1, which is a general amino acid transporter [53]). Thus, although melatonin is a ubiquitous molecule, its function in microorganisms is unknown. However, it has to be emphasised that in the organisms, where it has been studied, it exerts potent regulatory functions.

Melatonin can present up to nine isomers [54], including melatonin itself, because of the different pattern substitutions of the groups (*N*-acetyl-(2-aminoethyl)) and methoxy in the indolic ring. An isomer of melatonin was detected in wine. Indeed, the MS fragmentation ions of melatonin were different from those of the isomers found in wine [44]. Both melatonin and its isomer are present in different wine varieties, showing that those from the variety Jaen Tinto had the highest amount of melatonin isomer (21.9 ng/mL).

This finding was confirmed later by Gomez et al. [46], who described the isomer in Malbec wine and its formation during the fermentation step. Recently, Kocadağli et al. [55] detected the highest amount of melatonin isomer in yeast-fermented products (red wine, beer, and bread crump). Up to now, there is just one isomer of melatonin (*N*-acetyl-3-(2-aminoethyl)-6-methoxyindole) that is commercially available. However, when wines were spiked with this standard, the chromatograms showed 3 peaks with identical fragmentation pattern: melatonin, the* N*-acetyl-3-(2-aminoethyl)-6-methoxyindole, and the isomer not identified yet [44]. An analytical challenge is the position of the methoxy group in the indolic ring by mass spectrometry. Further analysis by NMR is required to elucidate its structure.

Serotonin is present at low levels in many plant-derived food products, including coffee beans, cherries, strawberries, and many others [56, 57]. Its role in the plant kingdom may be related to the regulation of root development probably by acting as a natural auxin inhibitor [58].

So far, there is no evidence for the production of serotonin by* S. cerevisiae*, although serotonin was some years ago found to be synthesised by yeast in response to UV radiation [59]. In fact, it is assumed that serotonin is an intermediate in the synthesis of melatonin in* Saccharomyces cerevisiae*, as it is in vertebrates [28, 60].

Serotonin is found in wines at levels ranging from 2 to 23 mg/L, mainly as a result of the malolactic fermentation, and significantly higher serotonin levels were observed when* Lactobacillus plantarum* was used [61, 62].

## 4. Conclusions

The metabolism of the aromatic amino acids in yeasts can produce a broad array of molecules that could be relevant from different aspects related to both yeast regulation and human health. The activities of these compounds as neurohormones and antioxidants open a new scenario of applications from nutrition supplements to functional foods. However, the role of these compounds in yeast is still far from being completely understood. Thus, it is still beyond our possibilities to modulate their production and their appearance in fermented food. Further research in the field of yeast metabolism related to the presence of aromatic amino acids would provide the theoretical basis for a broad array of applications in modern nutrition.

## Figures and Tables

**Table 1:** Ehrlich pathway intermediates and derivatives of aromatic amino acids.

Amino acid	Tryptophan	Tyrosine	Phenyl alanine
*α*-Keto acid	3-Indole pyruvate	p-Hydroxyphenyl pyruvate	Phenyl pyruvate
Higher aldehyde	3-Indole acetaldehyde	p-Hydroxyphenyl acetaldehyde	2-Phenylacetaldehyde
Higher alcohol	Tryptophol	Tyrosol	2-Phenylethanol
Higher acid	Indole acetic acid	4-Hydroxyphenyl acetic acid	Phenyl acetic acid
